# Primary epithelioid angiosarcoma originating from the mandibular gingiva: a case report of an extremely rare oral lesion

**DOI:** 10.1186/s12957-020-01999-1

**Published:** 2020-10-03

**Authors:** Yuko Komatsu, Ikuya Miyamoto, Yu Ohashi, Katsunori Katagiri, Daisuke Saito, Mizuki Obara, Yasunori Takeda, Kiyoto Shiga, Hiroyuki Yamada

**Affiliations:** 1 Division of Oral and Maxillofacial Surgery, Department of Oral and Maxillofacial Reconstructive Surgery, School of Dentistry, Iwate Medical University, 19-1 Uchimaru, Morioka, Iwate, 020-8505 Japan; 2 Head and Neck Cancer Center, Iwate Medical University, 2-1-1 Idaidori, Yahaba-cho, Shiwa-gun, Iwate, 028-3694 Japan; 3 Department of Head and Neck Surgery, School of Medicine, Iwate Medical University, 2-1-1 Idaidori, Yahaba-cho, Shiwa-gun, Iwate, 028-3694 Japan; 4 Division of Clinical Pathology, Department of Oral and Maxillofacial Reconstructive Surgery, School of Dentistry, Iwate Medical University, 2-1-1 Idaidori, Yahaba-cho, Shiwa-gun, Iwate, 028-3694 Japan

**Keywords:** Epithelioid angiosarcoma, Mandibular gingiva, Vascular tumor

## Abstract

**Background:**

Angiosarcoma occurs very rarely in the oral cavity, and the epithelioid type is even rarer. Here, we report a rare case involving an elderly man with a primary epithelioid angiosarcoma that originated from the mandibular gingiva and resembled a dentigerous cyst on radiographs.

**Case presentation:**

A 66-year-old Japanese man visited our hospital with a chief complaint of gingival swelling in right mandibular third molar region. A panoramic radiograph showed bone resorption around the crown of right mandibular third molar, which was impacted. Incisional biopsy confirmed a diagnosis of epithelioid angiosarcoma. The lesion exhibited aggressive proliferation after biopsy resulting in uncontrolled bleeding and difficulty in closing the mouth. Mandibular segmental resection including the tumor was performed without reconstruction. Because of the aggressive preoperative course of the tumor, the patient received adjuvant chemotherapy. There were no signs of recurrence during a 2-year follow-up period.

**Conclusions:**

A review of the literature yielded only four reported cases of epithelioid angiosarcoma in the jaw region, with the lesions occurring in the maxilla in three cases. To our knowledge, this is the second case of primary epithelioid angiosarcoma in the mandible.

## Background

Angiosarcomas account for only 2% of all soft tissue sarcomas [1–3]. More than 50% of all cases occur in the head and neck region, with the scalp and forehead being the most common sites [4]. However, primary angiosarcomas in the oral cavity represent only 1% of all angiosarcomas [5]. Angiosarcoma generally exhibits an aggressive clinical course and an unfavorable prognosis, even in cases where surgical removal of the tumor is possible. Affected patients usually do not benefit from chemoradiotherapy [6]. Approximately 50% patients die within 15 months after diagnosis, and only 12% survive for 5 years or longer [7]. Generally, well-differentiated tumors have been associated with better survival rates. Tumor-related death is usually due to uncontrolled local progression and/or distant metastases to the lungs, liver, and bone [1, 8]. Early detection and treatment are essential for controlling this highly malignant soft tissue tumor.

Epithelioid vascular neoplasm is a unique tumor characterized by proliferating “epithelioid” or “histiocytoid” endothelial cells showing a sheet-like growth pattern with vascular differentiation [9–11]. Epithelioid angiosarcoma (EA) is a rare variant characterized by the proliferation of atypical, large, polygonal, epithelioid endothelial cells [12].

Until now, only four cases of primary EA in the jaw region have been reported, with three lesions originating in the maxilla and one in the mandible [2, 11, 13, 14]. A few cases of metastatic EA have also been reported [15–17]. To our knowledge, there is no reported case of EA involving the mandibular gingiva except pathological review of EA by Nagata et al. [2]. Here, we report a case involving an elderly man with a primary EA that originated from the mandibular gingiva and resembled a dentigerous cyst on radiographs.

## Case presentation

A 66-year-old Japanese man visited a private dental office with a chief complaint of gingival swelling around right mandibular third molar. Considering the diagnosis of pericoronitis, the dentist performed incisional drainage and prescribed antibiotics and analgesics; however, the swelling reappeared with mild gingival bleeding 20 days after his first visit to the dental office. The dentist repeated incisional drainage and prescribed antibiotics, both of which were ineffective. The gingival swelling and oozing persisted; therefore, curettage of the swollen gingiva was performed. The dentist further referred the patient to our university hospital for extraction of the third molar, and he presented at our department 18 days after curettage of gingiva.

**Fig. 1:**
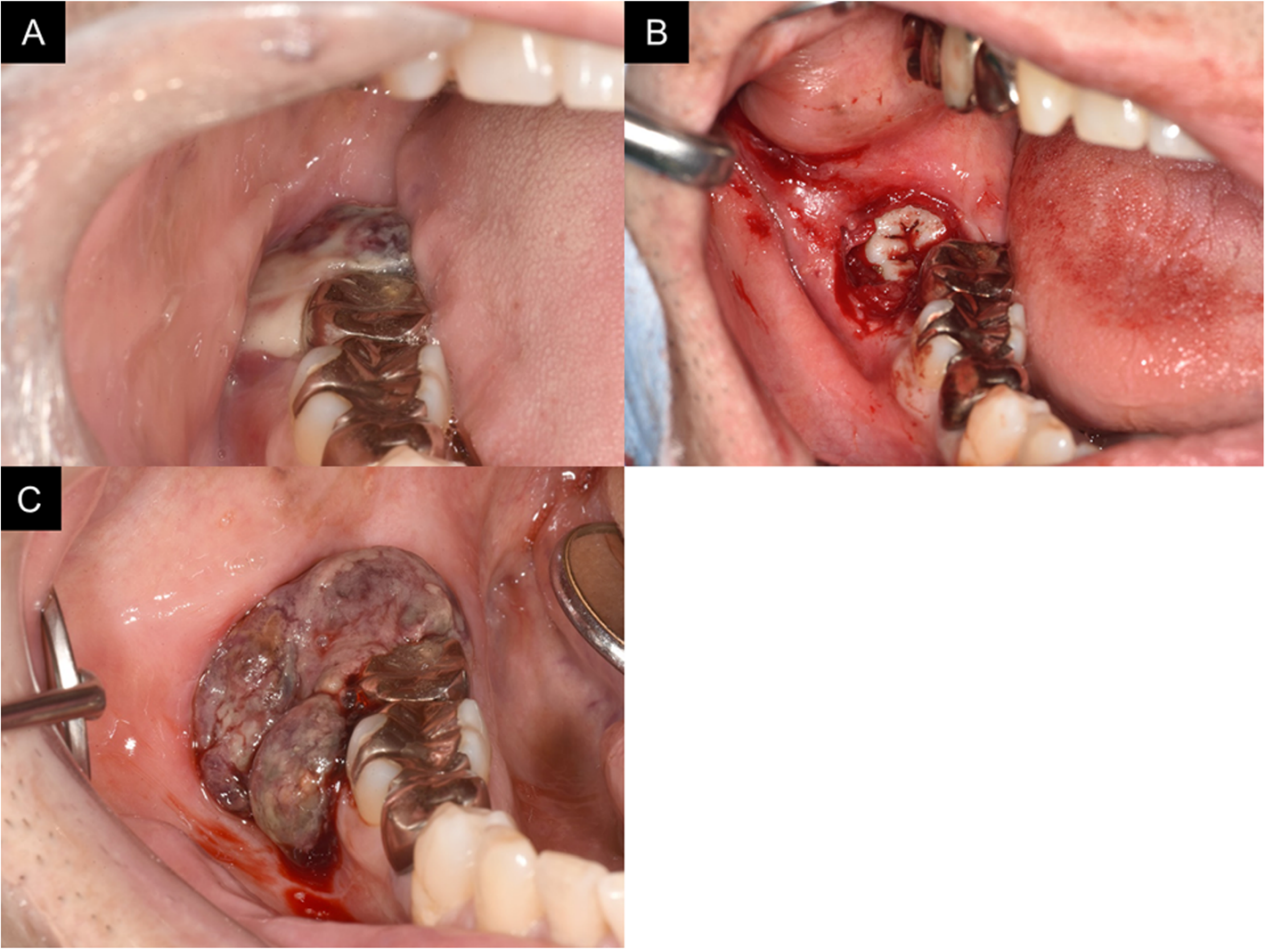
Intraoral views of the patient. **a** His gingiva was swollen covering the crown of the right mandibular third molar at his first visit to our outpatient clinic. **b** The crown of right mandibular third molar after gingival biopsy. His gingiva was fragile and easy to bleed. **c** The enlarged lesion 4 weeks after biopsy

**Fig. 2:**
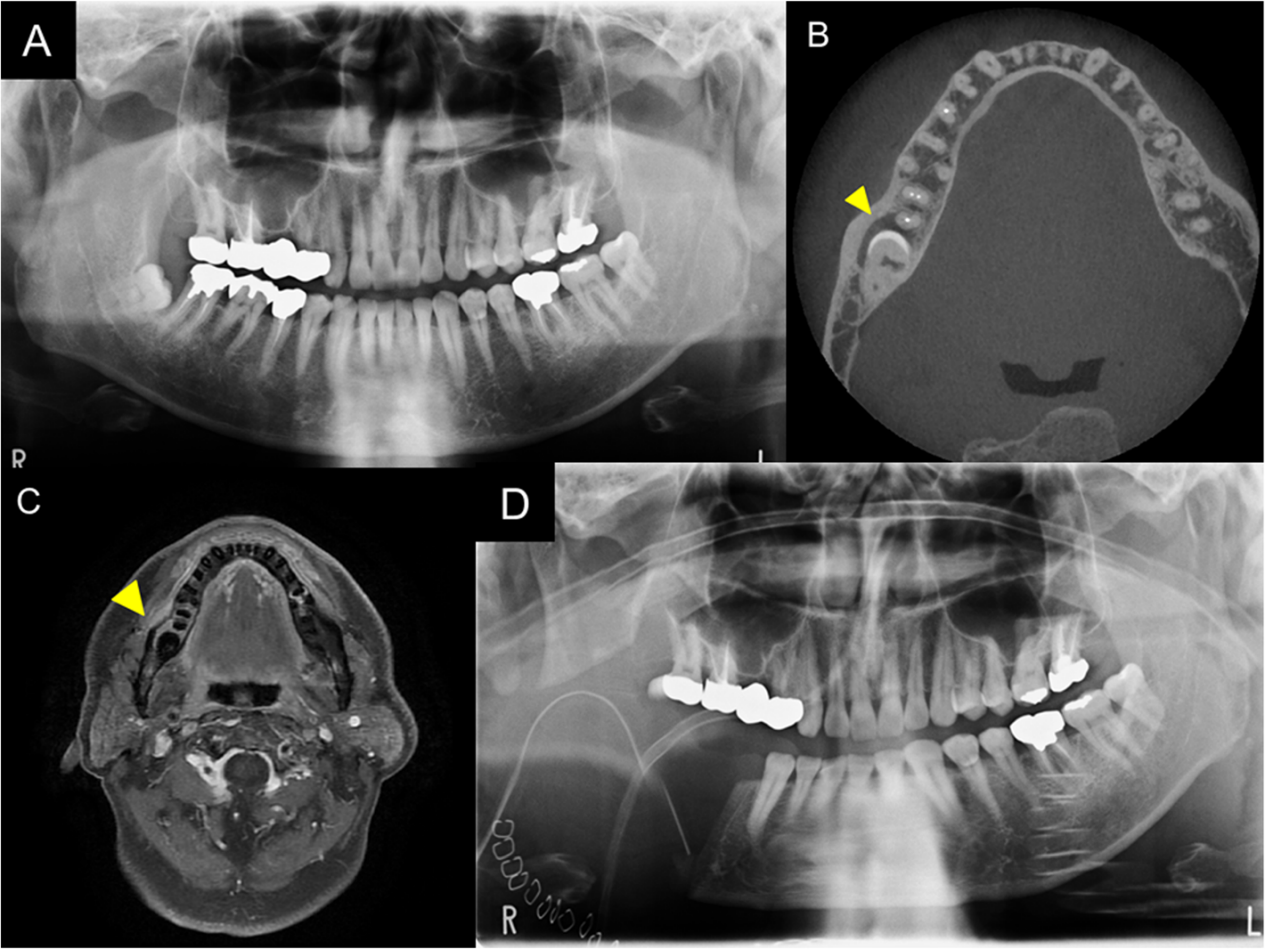
Images of the patient. **a** Panoramic radiograph at his first visit to our outpatient clinic, showing a small cystic lesion around the embedded crown of his right mandibular third molar. The lesion resembled a hyperplastic dental sac or a dentigerous cyst. **b** Cone beam computed tomography showing the tumor with mild bone consolidation around the crown of his right mandibular third molar (arrow head). **c** T2-wighted magnetic resonance imaging showing high signal intensity around the crown of his right mandibular third molar (arrow head). **d** Postoperative panoramic radiograph after segmental mandibular resection

The patient’s medical history included controlled hypertension and cholecystectomy for gallstones. Hematological and biochemical examinations did not reveal any abnormalities. His facial profile was symmetrical, and the submandibular lymph nodes showed no tenderness or swelling on ultrasound. Intraoral examination revealed a dark purple gingival swelling with easy bleeding around the crown of right mandibular third molar (Fig. 1a). The mass measured 1.5 × 1.5 cm and covered the tooth crown. A panoramic radiograph showed that right mandibular third molar was mesially impacted, with bone resorption around the crown (Fig. 2a). Considering the clinical course of the lesion, pericoronitis was ruled out and incisional biopsy was performed under local anesthesia (Fig. 1b). The specimen was submitted for microscopic examination. Histopathological examination showed an angiosarcoma. At the time of biopsy, cone beam computed tomography (CBCT) showed mild bone consolidation (Fig. 2b) while T2-weighted magnetic resonance imaging showed high signal intensity (Fig. 2c) around the involved tooth. The region to be imaged with gadolinium was recognized; this suggested inflammation after biopsy and/or malignancy. None of the imaging studies, including fluorodeoxyglucose (FDG) positron emission tomography-computed tomography, showed significant swelling of and accumulation of FDG in the cervical lymph nodes (data not shown).

Surgical treatment with vascularized free flap reconstruction was planned. However, 4 weeks after the biopsy, the lesion exhibited aggressive proliferation perhaps because of traumatic irritation by the antagonist teeth. This led to uncontrolled bleeding and difficulty in closing the mouth (Fig. 1c). The treatment plan was changed and emergent mandibular resection without surgical reconstruction was performed. An incision extending from the lower lip to the sub-mandible was made. A safety margin of ≥ 10 mm was set, a periosteal flap was raised, and the mandibular body was identified. Right mandibular first premolar was extracted to establish the anterior border of the resection. The sublingual gland was preserved and the tumor was resected en bloc with the oral floor membrane and buccal mucosa (Fig. 2d).

The patient’s postoperative course was uneventful except for a delayed wound healing of the oral mucosa. Nevertheless, because the tumor had exhibited an aggressive preoperative course, he received adjuvant chemotherapy 2 months after the surgery [18]. Radiation therapy was deemed unnecessary. Paclitaxel (80 mg/m^2^) was administered once a week for 3 weeks, followed by a 1-week rest period. This cycle was repeated for 1 year. The patient showed no signs of recurrence during the 1-year follow-up period.

**Fig. 3:**
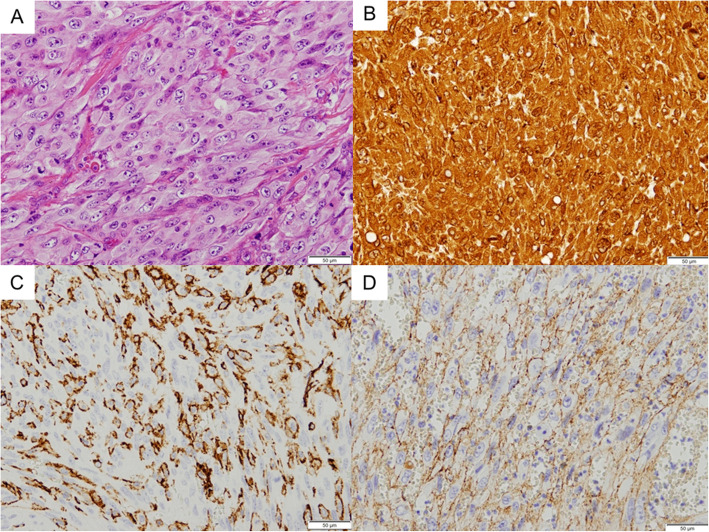
Histopathological examination. **a** Hematoxylin and eosin staining of the specimen showed large, densely packed cells with spindle, over, polygonal, or bizarre shapes. The cells had abundant amphophilic cytoplasm, large vesicular nuclei, and a few prominent nucleoli. Mitotic figures were scattered throughout the tumor (× 200). **b** Immunohistochemistry findings showing positivity for vimentin in the tumor cells (× 200). **c** Immunohistochemistry findings showing positivity for CD31 in the tumor cells (× 200). **d** Immunohistochemistry findings showing positivity for factor VIII in the tumor cells (× 200)

Histopathologically, the tumor was composed of solid sheets with incomplete alveolar structures (Fig. 3a) and no clear vasoformation. Large, densely packed cells with spindle, oval, polygonal, or bizarre shapes could be observed. The cells exhibited abundant amphophilic cytoplasm, large vesicular nuclei, and a few prominent nucleoli. Mitotic figures were scattered throughout the tumor. Keratinization and intercellular bridges were not obvious. Immunohistochemical analysis revealed positivity for vimentin (Fig. 3b), CD31 (Fig. 3c), and factor VIII (Fig. 3d) and negativity for epithelial, neural, muscular, and other markers. The Ki67 index was > 40% (Table 1). All of above the features are diagnostic for EA.

**Table 1:** Immunohistochemistry findings

**134βE12**	-
**AE1AE3**	**-**
**CAM5.2**	**-**
**CK5/6**	**-**
**P40**	**-**
**P63**	**-**
**EMA**	**-**
**E cadherin**	**-**
**NSE**	**-**
**CDK4**	**-**
**β catenin**	**-**
**S100**	**-**
**Melan-A**	**-**
**Desmin**	**-**
**Caldesmon**	**-**
**CD34**	**-**
**HHF35**	**-**
**Vimentin**	**+**
**CD31**	**+**
**Factor VIII**	**+**
**Ki67**	**> 40%**

## Discussion and conclusions

EA is a rare endothelial cell malignancy. Epithelioid endothelial cell tumors include epithelioid hemangioma, epithelioid hemangioendothelioma (EHE), and EA [14]. Epithelioid hemangioma is a benign tumor while EHE is a low-grade malignancy. EA is the least common type, and it is a high-grade malignancy characterized by atypical, multilayered, or solid endothelial proliferation and a vasoformative architecture on microscopic findings. EA displays a greater degree of nuclear pleomorphism and mitotic activity than does EHE and frequently shows areas of necrosis [19, 20]. Proposed criteria for distinguishing EA from other histopathological entities include a multilayered endothelium and an infiltrative growth pattern. Poorly differentiated angiosarcomas are best distinguished from other spindle cell sarcomas or carcinomas by immunohistochemistry. In particular, CD31 is the most sensitive endothelial marker [14, 19–21].

Angiosarcoma may be difficult to diagnose because of its various clinical and pathological characteristics [2]. Differential diagnoses include hemangioma, pyogenic granuloma, Kaposi’s sarcoma, melanoma, and metastatic disease [13, 14, 19, 20]. In the present case, primary EA of the mandibular gingiva was initially diagnosed as pericoronitis involving the mandibular third molar. In addition, CBCT suggested a hyperplastic dental follicle or a dentigerous cyst, and there were no signs of malignancy in imaging studies. Generally, radiographic findings for angiosarcoma may include osteolytic changes with a mild periosteal reaction [22]. Reviewing previous reports, it seems that the swelling of the gingiva accompanied by bone destruction with rapid growth of tumor (Table 2) [2, 11, 13–17]. In these cases, although the lesion may have originated from bone tissue, relatively well-defined margins may simulate a benign osteolytic lesion such as a cyst or an odontogenic tumor. There was a possibility that this tumor originated from bone tissue, however, the only clinical signs in the present case were gingival swelling and oozing, which is justified considering the origin of the tumor before osteolytic changes. The tumor enlarged rapidly after biopsy. Traumatic irritation by the antagonist teeth might accelerate the tumor growth, however, there is a possibility that this finding was due to the natural course of this rare aggressive tumor. Definitive diagnosis of angiosarcoma is challenging because of its rarity and clinical, radiographic, and histopathological resemblance to other diseases. Immunohistochemistry is generally required to identify this tumor, particularly the epithelioid and spindle cell types [2].

**Table 2:** Previous reports of epithelioid angiosarcoma. Four primary epithelioid angiosarcoma in the jaw region and three cases of metastatic epithelioid angiosarcoma have been reported

Author	Age	Sex	Primary	Metastases	Clinical oral findings	Clinical course
Fletcher et al. 1991 [15]	63	M	Buttock	Aorta, maxilla	Details unknown	No evidence of disease (30 months)
Freedman et al. 1992 [13]	32	M	Maxilla	-	Painless swelling of the left side of the hard palate	No evidence of disease (1.8 months)
Sasaki et al. 1996 [11]	69	M	Maxilla	Stomach, cerebrum, thoracic	Complaints of pain and swelling of the left maxilla	Died of the disease (9 months)
Triantafillidou et al. 2002 [14]	50	F	Maxilla	-	A painless swelling of the left maxilla	No evidence of disease (36 months)
Kawasaki et al. 2005 [16]	71	M	Scapula, Widespread	Mandible	Painless buccal gingival swelling in the left lower molar region	Died of the disease (1 month)
Peacock et al. 2014 [17]	64	M	Kidney	Mandibular condyle	Pain in the temporomandibular joint pain, and a malocclusion	No evidence of disease (30 months)
Nagata et al. 2014 [2]	55	M	Mandible	Thoracic, vertebrae	Rapidly expanding, bluish, hemorrhagic, and fragile mass	Died of the disease (9 months)
Present case 2020	66	M	Mandible	-	Rapidly expanding, bluish, hemorrhagic, and fragile mass	No evidence of disease (24 months)

In immunohistochemistry, positive staining for factor VIII, CD34, and CD31, which are endothelial markers, indicates that the tumor has endothelial characteristics. The lesion in our patient showed positivity for factor VIII, CD31, and vimentin. In particular, CD31, a membrane glycoprotein that is important for endothelial cell-cell interactions and vascular adhesion of leukocytes, is considered the best marker for the endothelial phenotype, especially the poorly differentiated variant [23]. Our patient also exhibited a very high Ki67 index (> 40%).

Most authors believe that surgery in combination with radiotherapy and/or chemotherapy offers the best chance of survival [14]. Angiosarcomas exhibit a strong tendency for local recurrence and metastasis [13]. These characteristics reflect the highly aggressive nature of this tumor. Our patient received postoperative adjuvant chemotherapy because of the aggressive preoperative course of the lesion. The prognosis of angiosarcoma is unfavorable, although the tumor size and site and the histopathological grade may influence survival [24]. In particular, a poor prognosis is associated with advanced age, larger tumors, the tumor location, and a Ki67 index of > 10% [25]. Radical surgery should be performed as soon as possible for improvement of the prognosis [2].

## Conclusions

We reported a rare case of a primary EA originating from the mandibular gingiva in an elderly man. To our knowledge, this is the first report of EA in the mandible, and the findings highlight the importance of early diagnosis and adequate surgical resection, which should be performed as soon as possible.

## Data Availability

The datasets used during the current study are available from the corresponding author on reasonable request.

## Abbreviations

EA: Epithelioid angiosarcoma
EHE: Epithelioid hemangioendothelioma
CBCT: Cone beam computed tomography
FDG: Fluorodeoxyglucose
